# An Unusual Case of Tooth in the Floor of the Orbit: The Libyan Experience

**DOI:** 10.1155/2012/954789

**Published:** 2012-09-30

**Authors:** Y. Naresh Shetty, Irfan Adil Majid, Raju Umaji Patel, Musbah Shammam

**Affiliations:** Department of Oral & Maxillofacial Surgery, Faculty of Dentistry, Melaka-Manipal Medical College, 75150 Melaka, Malaysia

## Abstract

Maxillary dentigerous cysts although uncommon need to be considered in the differential diagnosis in children with painless facial swelling. We present a case of dentigerous cyst associated with maxillary deciduous canine and maxillary premolars manifesting as a unilateral swelling in canine region of the face. A ten-year-old boy came to oral and maxillofacial surgery unit with a painless left facial swelling. The local dentist had prescribed antibiotics for treatment for the facial swelling, but the swelling did not subside, and the parents brought the child to our unit in Zliten Dental College. After clinical examination and imaging, the diagnosis of dentigerous cyst was made. Caldwell-Luc approach was done, the cyst was enucleated, and primary closure was done. The patient was followed up for a period of two years and there was no evidence of any recurrence.

## 1. Introduction

Cysts of the jaw usually present as asymptomatic swellings of the mandible and midface region. A dentigerous cyst is one that encloses the crown of an unerupted tooth by expansion of its follicle and is attached to its neck. In maxillary dentigerous cysts involving the canine region, extension into the maxillary sinus or to the orbital floor may be noted as seen in our case. Most dentigerous cysts are asymptomatic, and they are usually observed as an incidental finding on radiography. These cysts attain a large size with resorption of the roots of teeth till they manifest clinically or become evident radiographically. Treatment includes enucleation of the cyst with the removal of the unerupted tooth. Marsupialization is occasionally done with very large cyst to decompress the cyst. Prognosis is excellent with enucleation and recurrence is rare if the cysts is completely removed.

## 2. Case Report

**Figure 1:**
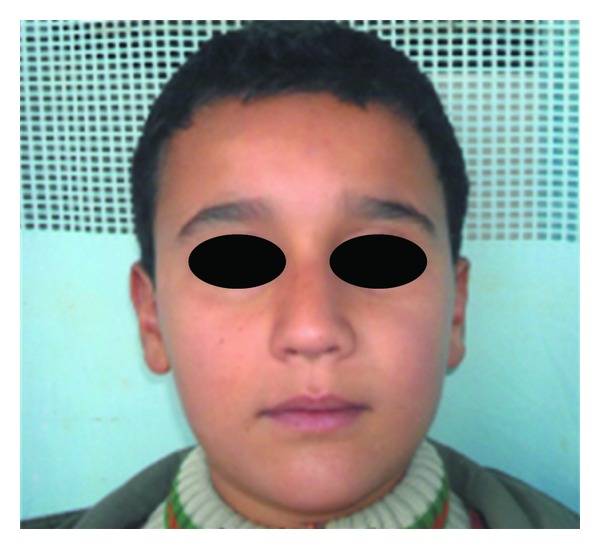
Preop Frontal View.

**Figure 2:**
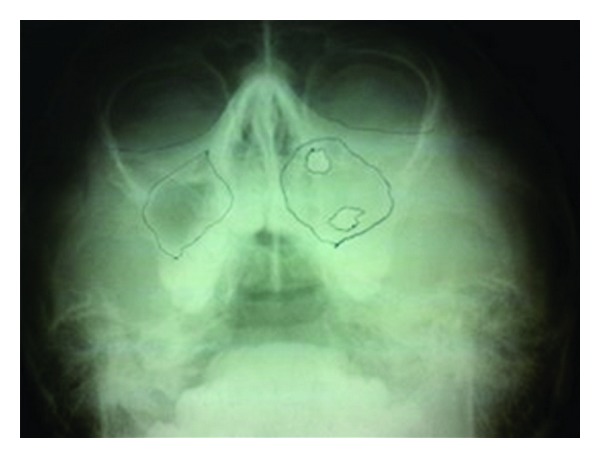
Paranasal sinus view.

**Figure 3:**
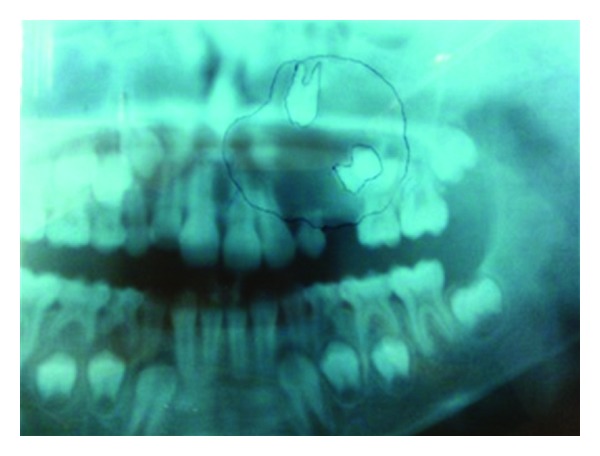
OPG View.

**Figure 4:**
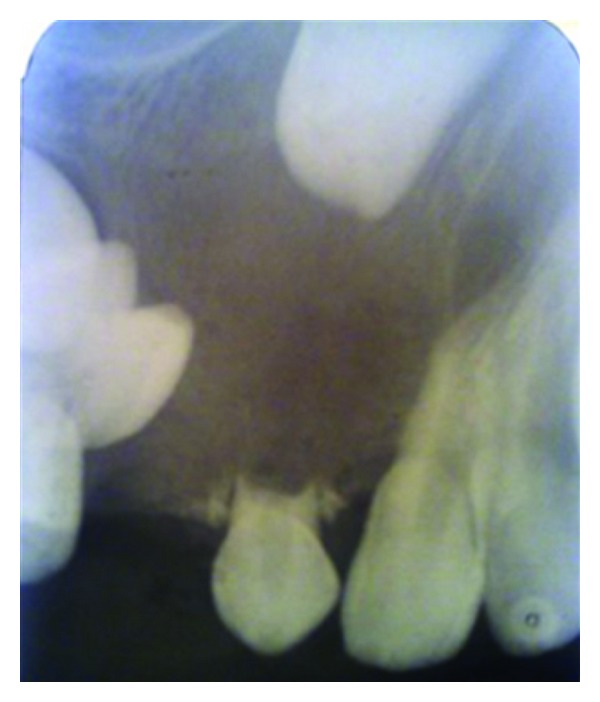
IOPA view showing deciduous canine and 1st premolar embedded in the cyst.

**Figure 5:**
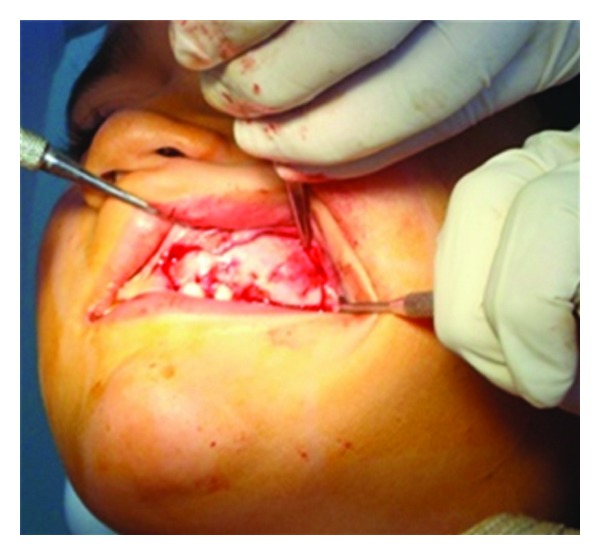
Intraoperative view.

**Figure 6:**
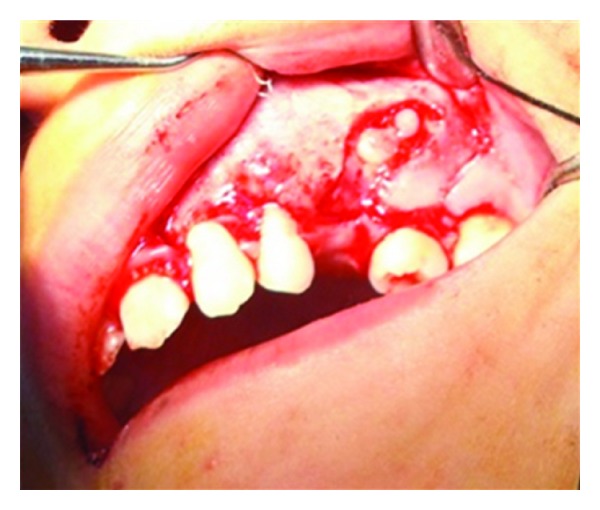
Canine tooth embedded within the cyst.

**Figure 7:**
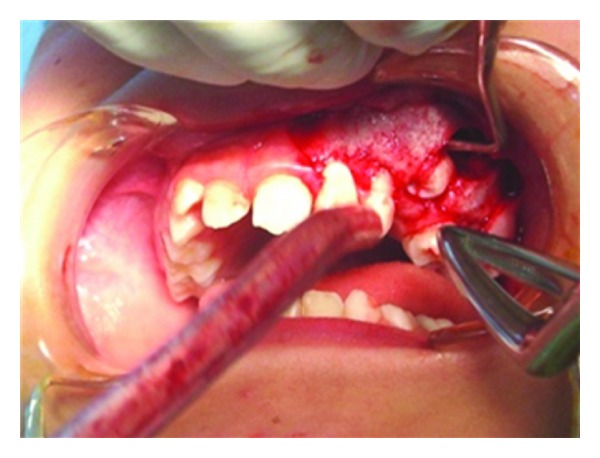
Premolar tooth in cyst.

**Figure 8:**
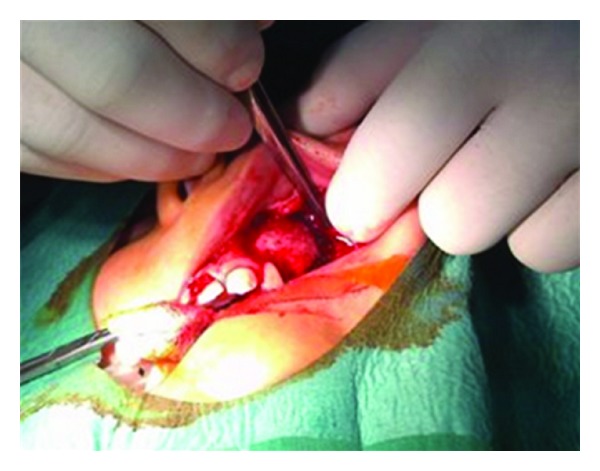
Complete cyst enucleation.

**Figure 9:**
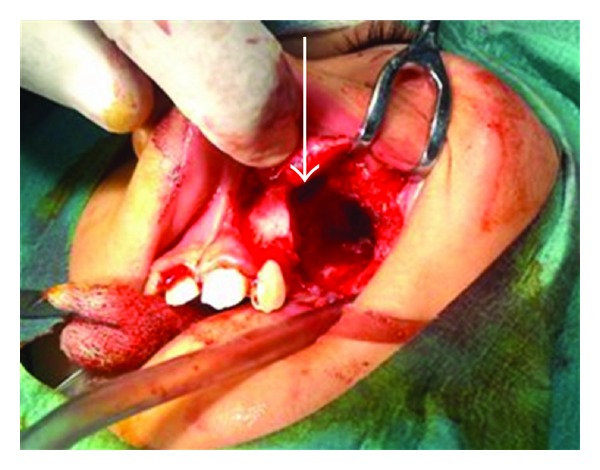
Cavity after enucleation arrow shows empty tooth socket in floor of the orbit.

**Figure 10:**
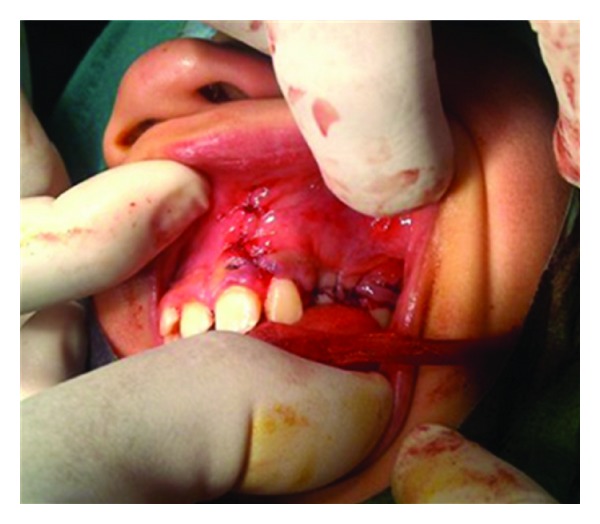
Primary closure done.

**Figure 11:**
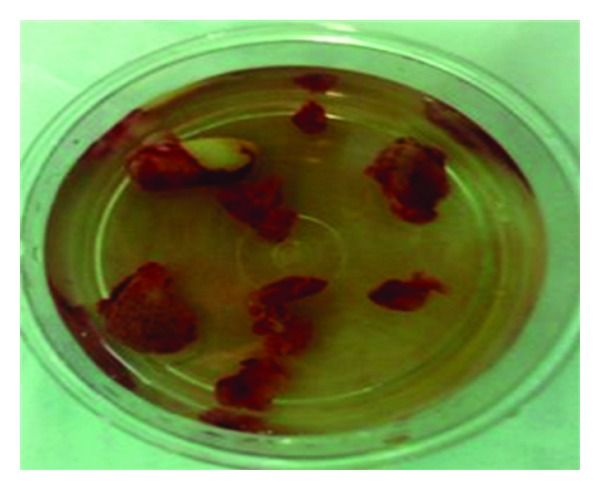
Biopsy specimen.

**Figure 12:**
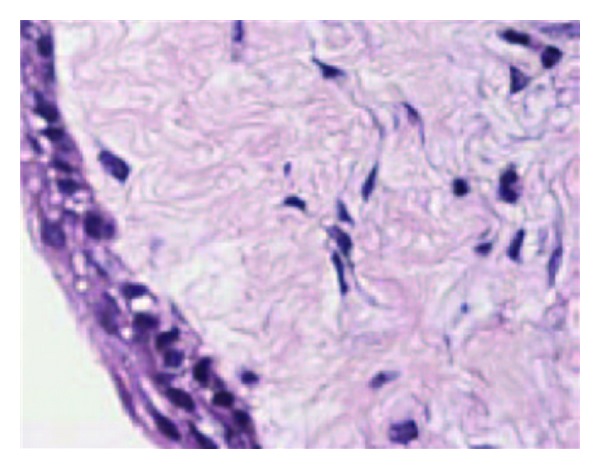
Histological slide.

**Figure 13:**
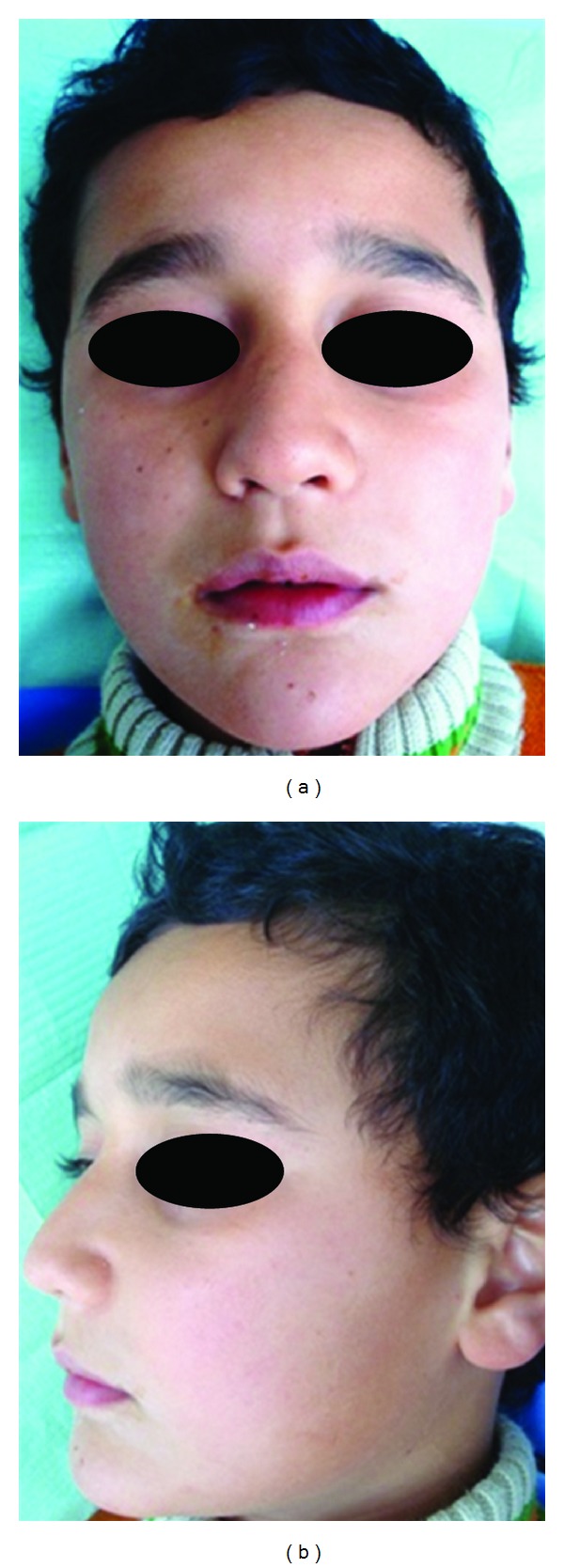
Postoperative frontal view and lateral view after 2 years.

A 10-year-old boy presented to our unit with the chief complaint of left facial swelling (Figure 1). A seven-day course of amoxicillin 250 mg thrice daily was prescribed by local dentist. At the next visit after one week, the mother expressed her concern as the swelling did not subside over the left maxillary sinus region. Neglect of the cyst usually causes expansion of cyst to produce impingement on surrounding structures like nasal septum, orbit, alveolar arch, and hard palate. Examination revealed a slight left facial swelling, which was nontender and firm, with no warmth, fluctuation, or discoloration of the overlying skin. The child was afebrile, in the rest of his physical examination, no abnormalities were seen. The swelling felt firm and “bony hard.” The child underwent imaging including intraoral periapical view, paranasal sinus view, and orthopantomogram (Figures 2, 3, and 4). The radiologic diagnosis was a large unilocular cyst associated with deciduous canine and premolar teeth. Caldwell-Luc approach was used and the cyst was enucleated (Figures 5, 6, 7, 8, 9, 10, and 11). The cyst measured 6.0-7.0–1.5 cm was excised, along with two teeth, first premolar and canine which were removed with the cyst lining. The specimen was sent to oral pathology department for histopathologic evaluation where multiple sections revealed a cyst lined by a nonkeratinized layer of stratified squamous epithelium (Figures 12 and 13). Therefore, the final diagnosis was a dentigerous cyst arising from canine and premolar tooth. The child did well postoperatively, and two weeks after the procedure the facial swelling had decreased considerably. The child was followed for a period of two years, with no recurrence. 

## 3. Discussion 

Dentigerous cyst is classified as a developmental cyst by the World Health Organization. The highest incidence of dentigerous cysts occurs during the second and third decade of life. The mandibular third molar and maxillary canines are involved most frequently. A greater incidence in males has been noted, with a ratio of 1.6 : 1 reported. They are the second most common type of odontogenic cyst and the most common developmental cyst of the jaws. In children from 2 to 14 years of age, dentigerous cysts account for 49% of intraosseous cystic lesions, with eruption cysts, odontogenic keratocysts, and radicular cysts accounting for more than 10% each. Approximately 24% of all true cysts in the jaw are dentigerous cysts [1]. However, most maxillary cysts arise as a result of defects in embryological development which occur either as abnormalities in the fusion of facial processes or abnormal development of the dental follicle. Dentigerous cyst is an epithelial-lined developmental cyst that encloses the crown of an unerupted tooth at the cementoenamel junction. The cyst arises from the separation of the follicle from the crown of an unerupted tooth. They can enlarge causing bone expansion and even pathologic fractures. The expansion of the dentigerous cyst is related to an increase in cyst fluid osmolality and the release of bone-resorbing factors. They are usually asymptomatic and are diagnosed on routine dental radiographs. Although it may involve any tooth, the mandibular third molars are the most commonly affected. However in cases where the canine is involved, the eruption of the maxillary canine is significantly related to the small size of the cyst and the patient's age [2]. Delayed eruption is the most common indication of dentigerous cyst formation. While a normal follicular space is 3 to 4 mm, a dentigerous cyst can be suspected, when the space is more than 5 mm. The cyst develops around the partially formed crown of a permanent tooth as a result of intrafollicular spread of periapical inflammation from an overlying diseased primary tooth. An extensive dentigerous cyst may surround the tooth or root and a fistula, either antrocutaneous or antrooral, can develop. Daley and pringle concluded from their study that from the local population, odontomas were by far the most common tumor (51.53%) followed by ameloblastomas (13.52%) and peripheral odontogenic fibromas (8.93%). Locally, radicular (periapical) cysts were the most common odontogenic cyst (65.15%) followed by the dentigerous cyst (24.08%) and the odontogenic keratocyst (4.88%). Only X-ray of the antra reveals a cyst or tooth or both. Foreign bodies, metallic and nonmetallic, may also lay dormant in the maxillary sinus [3]. Like other cysts, uncomplicated dentigerous cyst causes no symptoms, until the swelling becomes noticeable. It has also been reported in younger age, as in a 13 years old female by Shah et al. [4]. Main in his study showed that the pressure exerted by a potentially erupting tooth on an impacted follicle obstructs the venous outflow and thereby induces rapid transudation of serum across the capillary walls. The increased hydrostatic pressure of this pooling fluid separates the follicle from the crown, with or without the reduced enamel epithelium [5]. 

Because they are typically asymptomatic, dentigerous cysts are usually diagnosed on routine dental radiographs. The diagnosis of dentigerous cyst is based on a combination of radiographic and histological features. We took orthopantomogram, paranasal sinus view, and intraoral periapical radiographs for our case. Radiographically, a dentigerous cyst is presented as a well defined unilocular or occasionally multilocular radiolucency with corticating margins in association with the crown of an unerupted tooth. These cysts range in size from several millimeters to several centimeters, where they may compromise jaw bone integrity and produce facial asymmetry [6]. In the maxilla, dentigerous cysts may be destructive and may occupy the maxillary sinus; nasal cavities and even orbital encroachment may be observed as seen in our case. In cases of extensive bony involvement and presence of a complex cystic lesion, CT imaging becomes necessary. In our case, the cyst was unilocular, with canine, 1st premolar being embedded in the cyst as seen in radiographs in (Figures 2–4). CT imaging helps to rule out solid and fibroosseous lesions, displays bony detail, and gives exact information about the size, origin, content, and relationships of the lesions involving the maxilla [7]. Observation of the cortical plates and antral bony walls on CT helps to distinguish an antral from an extra-antral maxillary lesion. MRI may fail to show the bony detail but precisely displays the lesional contents and provides information about the cyst fluid. The cystic lesions appear homogeneously hypointense on T1-weighted images and hyperintense on T2-weighted images [8]. Dentigerous cysts cause expansion and remodeling of sinus walls if they involve the maxillary sinuses, and the impacted tooth lies eccentrically in the cyst wall, appearing hypo intense on all sequences. Within the sinus, chronic secretions, air, acute hemorrhage, and mycetoma may also appear hypo intense, but their central location in the sinus distinguishes them from teeth. Surgical excision and pathologic analysis of the lesion is essential for the definitive diagnosis. Maxillary cysts may displace and obliterate the maxillary antrum and nasal cavities. The cysts may cause fractures and become secondarily infected. Metaplastic and dysplastic changes may occur. An ameloblastoma, mucoepidermoid carcinoma, or squamous cell carcinoma may develop from the lining epithelium of a dentigerous cyst. Associated aneursmal bone cysts and hemangiomas have been reported in rare instances. Most dentigerous cysts are solitary. Dentigerous cysts are known to recur rarely [9].

## 4. Treatment

Dentigerous cysts are frequently treated surgically, either by enucleation or marsupialisation. Following enucleation of the cyst and extraction of the unerupted tooth, the prognosis is excellent and recurrence is rarely observed after a complete removal. Surgical enucleation combined with the Caldwell-Luc approach followed by primary closure is recommended in treatment of the large maxillary sinus cyst as marsupialisation of these cysts towards the oral cavity will consequently create an oroantral fistula [10]. In our case, we performed a Caldwell-Luc procedure with enucleation of the cyst as the cyst was large and the ectopic tooth lies more medially in the floor of the orbit (Figures 5–11). Also it is evidenced from the study that it is not necessary to perform antrostomy at the inferior meatus for Caldwell-Luc procedure for odontogenic pathology [11]. If the tooth is in a favorable position and space is available, it may occasionally possible to marsupialise a dentigerous cyst to allow the tooth to erupt. In our case, as the tooth was displaced to orbital floor far away from alveolar arch with a questionable viability and is unlikely to erupt on its own, we enucleated the cyst with removal of the displaced tooth in the floor of the orbit. The patient was followed up for two years with no recurrence of the cyst. 

## 5. Conclusion

Our patient presented with a painless left facial swelling. Over a period of several weeks, the lesion expanded and caused left facial deformity. Additional imaging and histological examination confirmed the diagnosis of dentigerous associated with a canine and first premolar tooth. Although dentigerous cysts are more often found in the mandible, they may occur in the maxilla and rarely may infiltrate the floor of the orbit as seen in our case. Caldwell-Luc approach and enucleation of the cyst have been the mainstay of maxillary sinus surgery over the past century and still prove to be the method of choice.
